# Catalytic Performance of a Magnetic Core-Shell Iron(II) C-Scorpionate under Unconventional Oxidation Conditions

**DOI:** 10.3390/nano10112111

**Published:** 2020-10-23

**Authors:** Inês A. S. Matias, Ana P. C. Ribeiro, Ana M. Ferraria, Ana M. Botelho do Rego, Luísa M. D. R. S. Martins

**Affiliations:** 1 Centro de Química Estrutural and Departamento de Engenharia Química, Instituto Superior Técnico, Universidade de Lisboa, 1049-001 Lisboa, Portugal; ines.matias@tecnico.ulisboa.pt; 2 BSIRG, IBB-Institute for Bioengineering and Biosciences, Departamento de Engenharia Química, Instituto Superior Técnico, Universidade de Lisboa, 1049-001 Lisboa, Portugal; ana.ferraria@tecnico.ulisboa.pt (A.M.F.); amrego@tecnico.ulisboa.pt (A.M.B.d.R.)

**Keywords:** magnetic catalyst, core-shell, iron, magnetic nanoparticles, alcohol oxidation, alcohol dehydration, sonochemistry, microwave, ultrasound, mechanochemistry

## Abstract

For the first time, herein is reported the use of a magnetic core-shell support for a C-scorpionate metallic complex. The prepared hybrid material, that consists on the C-scorpionate iron(II) complex [FeCl_2_{κ^3^-HC(pz)_3_}] (pz, pyrazolyl) immobilized at magnetic core-shell particles (Fe_3_O_4_/TiO_2_), was tested as catalyst for the oxidation of secondary alcohols using the model substrate 1-phenylethanol. Moreover, the application of alternative energy sources (e.g., ultrasounds, microwaves, mechanical or thermal) for the peroxidative alcohol oxidation using the magnetic heterogenized iron(II) scorpionate led to different/unusual outcomes that are presented and discussed.

## 1. Introduction

Composite core-shells are highly functional materials. All micro- or nano-particle constituted of either an internal particle (core) or a set of internal particles (multicore), and a simple or multiple coating material (shell) of different nature, are designated core-shell structures [1]. They often possess enhanced chemical and physical properties (e.g., high surface area, reactivity or thermal stability) compared to the single components [2]. In fact, coating particles with a thin shell of a compatible material allows controlling surface properties and/or introduce desirable physical and chemical characteristics (according to the nature of the coating material), thereby expanding to a broader range of potential use [3].

Inorganic core-shell particles find applications in many fields [4] as a result of their multiple functions (e.g., optical, magnetic, catalytic) [5]. In this work magnetite (Fe_3_O_4_) and titanium oxide (TiO_2_) were chosen as core and shell, respectively, as they combine several advantageous features. Magnetite are magnetic nanoparticles (MNPs) resulting from nanomaterial technology [4,6] that exhibits benefits in several applications. However, Fe_3_O_4_ nanoparticles easily experience agglomeration. Thus, it is necessary to functionalize them by coating through, e.g., titanium oxide (TiO_2_) which is stable, non-toxic and relatively inexpensive [7,8].

**Figure 1:**
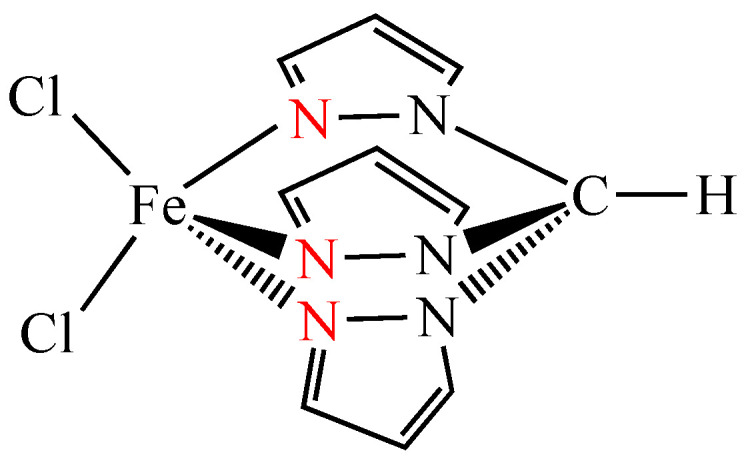
Structure of the C-scorpionate iron(II) complex [FeCl_2_{κ^3^-HC(pz)_3_}] (pz, pyrazolyl).

At core-shell composites, new processes can occur at the interface between the catalyst and the supporting material. For example, ferroelectric nanoparticles may lead to electric polarization at the interface with catalytic active metallic complexes. In addition, magnetic systems are easy to be conveniently fixed, separated and collected by an external magnet [9]. This possibility is a significant operational advantage as the usually required separation techniques (e.g., filtration or centrifugation), leading to high catalyst losses, can be avoided. In this work, the C-scorpionate iron(II) complex [FeCl_2_{κ^3^-HC(pz)_3_}] (pz, pyrazolyl, Figure 1) was chosen due to its recognized catalytic activity and versatility in heterogenization procedures and materials [10,11,12,13,14,15,16,17]. 

The hybrid material consisting on the C-scorpionate iron(II) complex [FeCl_2_{κ^3^-HC(pz)_3_}] (pz, pyrazolyl) immobilized at multiwalled carbon nanotubes (MWCNT), [FeCl_2_{κ^3^-HC(pz)_3_}]@MWCNT, was already recognized [18] as catalyst for the microwave-assisted oxidation of 1-phenylethanol by *tert*-butyl hydroperoxide (TBHP, 70% aqueous solution). The magnetization of [FeCl_2_{κ^3^-HC(pz)_3_}] by dry milling treatment with ferrite was also successfully achieved [19]. However, to our knowledge, the immobilization of this iron(II) complex at magnetic core-shell particles was never attempted.

The preference for 1-phenylethanol as a model substrate arises from the significance of the oxidized product (acetophenone) widely used as feedstock to synthesize several valuable chemicals (including insecticides, pharmaceuticals, and resins) and which is industrially produced via the non-sustainable oxidation of ethylbenzene [20,21].

In addition, the knowledge on how iron-containing catalytic materials would respond to inductive heating or mechanical stimuli (e.g., to ultrasounds) is still scarce [22,23]. Thus, it would be interesting to find routes for the sustainable synthesis of particulate iron-containing materials and study their catalytic performance under different energy inputs, such as ultrasound, microwave or oscillating magnetic irradiations. 

In this work, a C-scorpionate iron(II) complex supported at a magnetic core-shell Fe_3_O_4_/TiO_2_ was, for the first time, successfully prepared and its catalytic performance studied in the oxidation of 1-phenylethanol under different energy inputs (mechanical, thermal, sonication and microwave irradiation).

## 2. Materials and Methods 

### 2.1. Materials and Instrumentation

All reagents were purchased from Sigma-Aldrich (Munich, Germany) and used as received. Solvents were purified, when necessary, by standard methods and freshly distilled under dinitrogen immediately prior to use. The chloro C-scorpionate iron(II) complex [FeCl_2_{κ^3^-HC(pz)_3_}] (pz, pyrazolyl) was prepared and characterized according to a published method [24].

The synthesized core-shell particles, before and after functionalization with [FeCl_2_{κ^3^-HC(pz)_3_}], were characterized by Fourier transform infrared spectroscopy (FTIR), X-ray Photoelectron spectroscopy (XPS), X-ray diffraction (XRD), thermogravimetric analysis (TGA), scanning electron microscope (SEM) and energy dispersive spectroscopy (EDS). XPS analyses were performed using a XSAM800 spectrometer (KRATOS, Manchester, UK) with non-monochromatic Al Kα X-radiation (*hν* = 1486.6 eV). Operating conditions, data collection and processing details are described elsewhere [19]. The charge shifts were corrected using the aliphatic carbon binding energy centered at 285 eV as reference. The sensitivity factors were: 0.278 for C 1s, 0.78 for O 1s, 0.891 for Cl 2p, 0.477 for N 1s, 2.957 for Fe 2p and 2.001 for Ti 2p. TGA measurements were run on a STA 6000 (PerkinElmer, Boston, MA, USA). Morphology and distribution of the core shells and the catalyst were characterized using a SEM (JEOL 7001F with Oxford light elements EDS detector and EBSD detector, JEOL, Tokyo, Japan). FTIR analyses were performed on a Bruker Vertex 40 Raman/IR spectrometer (Billerica, MA, USA) in a range from 4000 to 100 cm^−1^. X-ray diffraction analysis were made in a Bruker D8 ADVANCE Powder Diffractometer (Bruker Corporation, Billerica, MA, USA), with Cu radiation in a Bragg Brentano geometry. The iron content of the core-shell supported catalyst was analyzed by inductively coupled plasma atomic emission spectroscopy (ICP-AES) carried out by Laboratório de Análises of IST using an ICP-AES model Ultima (Horiba Jobin-Yvon, Kyoto, Japan) apparatus.

Catalytic reactions under microwave (MW) irradiation were performed in a focused Anton Paar Monowave 300 reactor (Anton Paar GmbH, Graz, Austria) fitted with an IR temperature detector, and a rotational system, in a Pyrex cylindrical tube (10 mL capacity, 13 mm internal diameter). Sonochemistry was performed using a Sonoplus HD2200 sonicator (Bandelin, Berlin, Germany). Several reactions were mechanically performed using a high-energy ball mill (mixer mill MM 500 nano, Retsch, Haan, Germany), which generates an active surface contact by combining impact and shear stress.

Gas chromatographic (GC) experiments were run at a FISONS Instruments GC 8000 series gas chromatograph equipped with a flame ionization (FID) detector and a capillary column (DB-WAX, column length of 30 m; column internal diameter of 0.32 mm) and run by the software Jasco-Borwin v.1.50 (Jasco, Tokyo, Japan). He was the carrier gas. The temperature of injection was 240 °C. After injection, the reaction temperature was maintained at 140 °C for 1 min, then raised, by 10 °C/min, either to 220 °C and held for 1 min at this temperature. Gas chromatography-mass spectrometry analyses were carried out at a Perkin Elmer Clarus 600 C (Shelton, CT, USA) instrument (He as the carrier gas), having two capillary columns (SGE BPX5; 30 m × 0.32 mm × 25 mm), one having an electron impact (EI-MS) detector and the other one with a FID detector. All products were identified by comparing their retention times with known reference compounds as well as their mass spectra to fragmentation patterns obtained from the NIST spectral library of the mass spectrometer.

### 2.2. Synthesis of Magnetic Fe_3_O_4_

The co-precipitation method was used. To a round-bottom flask with (NH_4_)_2_Fe(SO_4_)_2_ · 6H_2_O and FeCl_3_ dissolved in water at 50 °C and 600 rpm, 1 M NaOH was added dropwise until pH 10 is reached. After cooling to room temperature, the supernatant was decanted and the magnetic residue was washed with H_2_O and ethanol (EtOH). The magnetic Fe_3_O_4_ solid was dried in an oven at 80 °C.

### 2.3. Synthesis of Magnetic Core-Shell Fe_3_O_4_/TiO_2_

100 mg of synthetized Fe_3_O_4_ (Section 2.2) were added to 150 mL of dry ethanol, 2 mL of titanium(IV) isopropoxide and distilled water. The mixture was heated to reflux with stirring for 2 h. After cooling to room temperature, the liquid phase was separated from the deposited particles, with the help of a magnet. The obtained magnetic core-shell Fe_3_O_4_/TiO_2_ particles were then taken to dryness under vacuum overnight at 40 °C.

### 2.4. Synthesis of Magnetic Fe_3_O_4_/TiO_2_/[FeCl_2_{κ^3^-HC(pz)_3_}]

18.3 mg of [FeCl_2_{κ^3^-HC(pz)_3_}] were added to 150 mg of the support (previously synthetized core-shell Fe_3_O_4_/TiO_2_; Section 2.3) in 25 mL of distilled water. The mixture was stirred for 24 h. Then, the composite was separated by filtration and washed several times with water. A silver nitrate solution was used in order to confirm the presence of chlorides. Once the formation of a white precipitate in the solution disappears, it means that the particles are already sufficiently washed. Finally, the immobilized material was washed with methanol and left to dry in the vacuum line at 40 °C to constant weight.

### 2.5. Oxidation of 1-Phenylethanol

In general the reaction mixtures were prepared as follows: 30 mg (1.56 μmol relative to the scorpionate complex) of Fe_3_O_4_/TiO_2_/[FeCl_2_{κ^3^-HC(pz)_3_}] were added to 605 µL (5 mmol) of 1-phenylethanol (substrate) and 1380 µL (10 mmol) of *tert*-butyl hydroperoxide (TBHP, 70% aqueous solution, oxidant). All reactions were run in a solvent-free system.

Recyclability of the magnetic catalyst, when applicable, was investigated through its recover and reuse in consecutive catalytic cycles. A new cycle was initiated after the previous one, by addition of new portions of substrate and oxidant. Catalyst recovery was achieved by using an external magnetic field. The products were analyzed as above-mentioned after completion of each run. The recovered catalyst was washed with acetonitrile and dried in an oven at 50 °C until constant weight. Iron leaching from the composite was evaluated throughout the determination of the iron content of the recovered solid by ICP-AES.

#### 2.5.1. Oxidation of 1-Phenylethanol Using Conventional Thermal Heating

The catalytic tests were performed in a batch reactor operated under atmospheric conditions at the chosen temperature (from 80 to 120 °C) for the established time (from 60 to 180 min). Then, the mixture was cooled down to room temperature. Due to the magnetic nature of the core–shell composite, it was easily separated from the reaction mixture by using a magnet. To conduct the product analysis, nitromethane (internal standard, 50 μL) and 1 mL of acetonitrile (MeCN) were added to 100 µL of the reaction mixture. Then, the sample was centrifuged for 15 min and an aliquot (1 µL) was taken from the organic phase and analyzed by GC.

#### 2.5.2. Microwave-Assisted Oxidation of 1-Phenylethanol

The focused microwave irradiation of the reactional mixture was performed with stirring for 10–180 min at the desired temperature (from 80 to 120 °C). After the reaction, the mixture was cooled to room temperature. Due to its magnetic nature, the core–shell composite was easily separated from the liquid by using an external magnetic field. Then the mixture was centrifuged and filtrated to prepare the samples for GC analysis. Acetonitrile (1 mL) and the internal standard (nitromethane, 50 μL) were added. The obtained mixture was stirred for 5 min and then a sample (1 µL) was taken from the organic phase and analyzed by GC.

#### 2.5.3. Ultrasounds-Assisted Oxidation of 1-Phenylethanol

A similar procedure to the one described at Section 2.5.2 was performed. A sonicator, instead of a microwave reactor, was used at room temperature.

#### 2.5.4. Mechanochemical Oxidation of 1-Phenylethanol

To perform the oxidation under mechanochemical (ball-milling) treatment, the catalytic composite (see Section 2.5) was directly added to the ball mill grinding bowl having 3 spheres, where after the substrate (1-phenylethanol) and the oxidant (TBHP, 70% aqueous solution) were added at room temperature. The ball mill was set at 35 Hz for the desired time (from 60 to 180 min). After the reaction, the mixture was left to cool down to room temperature and the core–shell composite was collected under the action of an external magnetic field. Then, acetonitrile (1 mL) and the internal standard (nitromethane, 50 μL) were added and the mixture was centrifuged. An aliquot (1 µL) was taken from the organic phase and analyzed by GC.

## 3. Results

Magnetic core–shell Fe_3_O_4_/TiO_2_ particles were prepared by seed mediated growth of semiconductor (TiO_2_) through a modified sol-gel process at preformed magnetite (Fe_3_O_4_) cores obtained by the co-precipitation method. Their full characterization was achieved by standard spectroscopic means, scanning electron microscopy (SEM), thermogravimetry-differential scanning calorimetry (TG-DSC), X-ray diffraction and X-ray photoelectron spectroscopy (see below).

**Figure 2:**
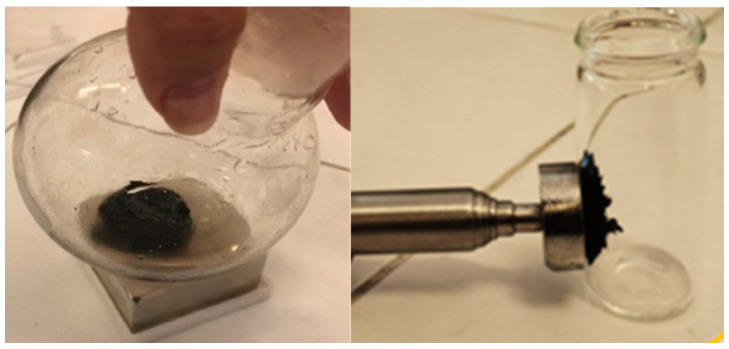
Synthesized magnetic core-shell Fe_3_O_4_/TiO_2_/[FeCl_2_{κ^3^-HC(pz)_3_}] composite.

Then, the freshly synthesized [24] C-scorpionate iron(II) complex [FeCl_2_{κ^3^-HC(pz)_3_}] was immobilized onto the above Fe_3_O_4_/TiO_2_ core-shells and the resulting Fe_3_O_4_/TiO_2_/[FeCl_2_{κ^3^-HC(pz)_3_}] composite (see Figure 2) fully characterized using the above-mentioned techniques. The iron loading of 0.3 wt% was achieved (Fe content determined by inductively coupled plasma atomic emission spectroscopy, ICP-AES).

### 3.1. X-ray Photoelectron Spectroscopy (XPS)

**Figure 3:**
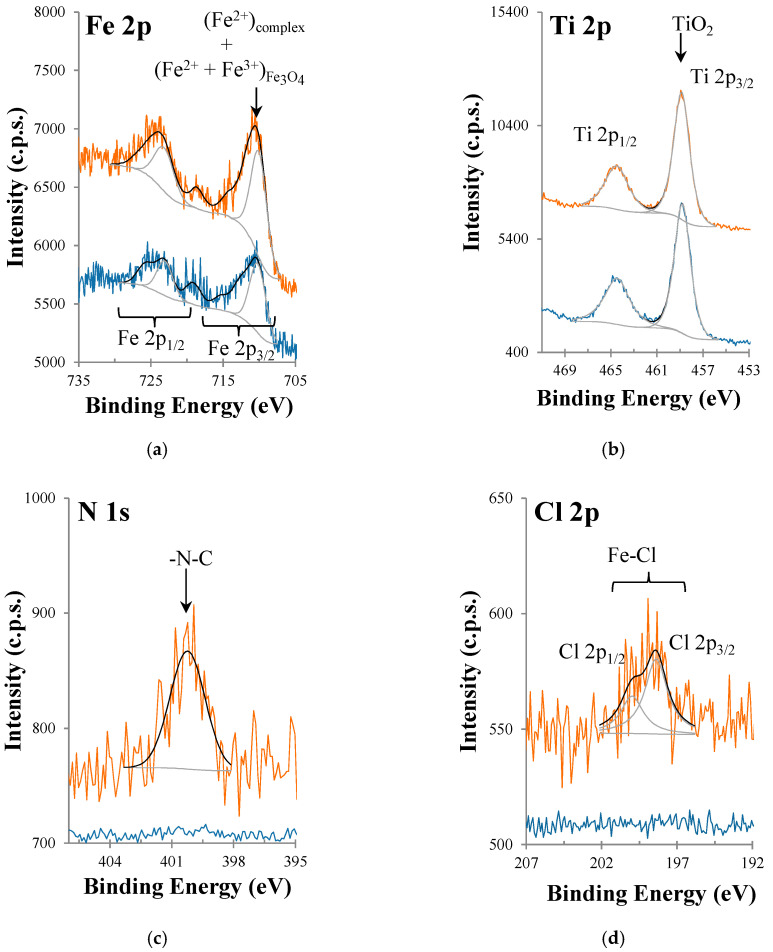
X-ray Photoelectron spectroscopy (XPS) regions: (**a**) Fe 2p; (**b**) Ti 2p; (**c**) N 1s; (**d**) Cl 2p of Fe_3_O_4_/TiO_2_ (bottom) and Fe_3_O_4_/TiO_2_/[FeCl_2_{κ^3^-HC(pz)_3_}] (top).

Figure 3 compares the relevant XPS regions of the structures Fe_3_O_4_/TiO_2_ and Fe_3_O_4_/TiO_2_/[FeCl_2_{κ^3^-HC(pz)_3_}]: Fe 2p, Ti 2p, N 1s and Cl 2p.

Doublets fitted in Fe 2p region (Figure 3a) have spin-orbit separations of 13.1 ± 0.2 eV (only fitted main doublets are shown). Fe 2p_3/2_ component in Fe_3_O_4_/TiO_2_ centered at 710.2 eV includes Fe^2+^ and Fe^3+^ contributions of the iron oxide. In Fe_3_O_4_/TiO_2_/[FeCl_2_{κ^3^-HC(pz)_3_}], this component, centered at 710.0 eV, also includes Fe^2+^ from the metal complex, whose presence is undoubtedly attested by N 1s and Cl 2p regions (Figure 3c,d) [19]. The most intense component in Ti 2p centered at 458.8 ± 0.1 eV is attributed to Ti^4+^. Since the titanium oxidation state in TiO_2_ is the same as in its precursor (titanium(IV) isopropoxide) only a quantitative analysis can attest its assignment to TiO_2_: the experimental atomic ratios CH_3_/Ti = 1.33 and (C bound to O)/Ti = 0.26, computed from C 1s and Ti 2p regions, show that the major part of the precursor (where the nominal atomic ratios CH_3_/Ti and C-O/Ti are, respectively, 8 and 4) had reacted under the conditions described above, producing TiO_2_. Moreover, the experimental atomic ratio Ti/Fe = 6.1 is compatible with a core-shell structure where TiO_2_ envelopes a core of Fe_3_O_4_, otherwise, if a homogeneous mixture of Fe_3_O_4_ and TiO_2_ existed, the atomic ratio Ti/Fe would be equal to 2.6 (this ratio was computed taking in account the synthesis details described in the experimental section).

### 3.2. Thermogravimetric Analysis (TGA)

**Figure 4:**
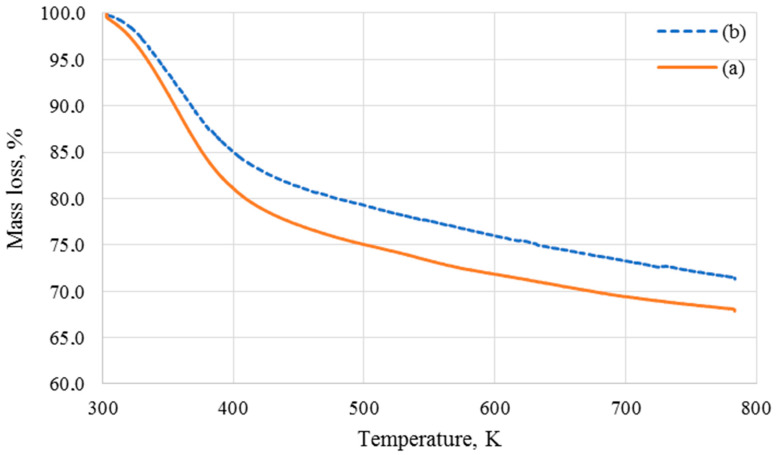
TGA for the: (**a**) magnetic core-shells Fe_3_O_4_/TiO_2_; (**b**) Fe_3_O_4_/TiO_2_/[FeCl_2_{κ^3^-HC(pz)_3_}].

TGA was performed to detect changes in chemical and/or physical properties of the magnetic core-shell materials as function of temperature variation. Figure 4 shows the TGA curves of Fe_3_O_4_/TiO_2_ and Fe_3_O_4_/TiO_2_/[FeCl_2_{κ^3^-HC(pz)_3_}] composites where a weight loss of ca. 15% and 20% is found for Fe_3_O_4_/TiO_2_/[FeCl_2_{κ^3^-HC(pz)_3_}] and Fe_3_O_4_/TiO_2_, respectively, up to 373 K. This might be due to the evaporation of physisorbed water molecules on the surface. Then, until 783 K, a 15% and 10% mass loss for Fe_3_O_4_/TiO_2_/[FeCl_2_{κ^3^-HC(pz)_3_}] and Fe_3_O_4_/TiO_2_ was detected. The higher percentage loss from the sample with the immobilized scorpionate iron complex is attributed to the loss of surface moieties. No other weight losses were observed. In addition, as expected, at the end of the analysis, the composites maintained their magnetic character.

### 3.3. Scanning Electron Microscopy (SEM) and Energy Dispersive X-ray (EDX)

The structure and morphology of prepared magnetic materials were characterized by SEM-EDX. Rugose microparticles (size ca. 300 µm) exhibiting irregular morphology were formed by coating magnetite-rich powder with TiO_2_, Figure 4a. Since the density of TiO_2_ is considerably lower than that of magnetite, the percentages of titania incorporated in the solid represent an important covering volume (shell). It is worth to mention that a comparison with literature data would be difficult as the percentages of titania incorporated in core-shells are not usually mentioned. Instead, literature reports the thickness of shells.

**Figure 5:**
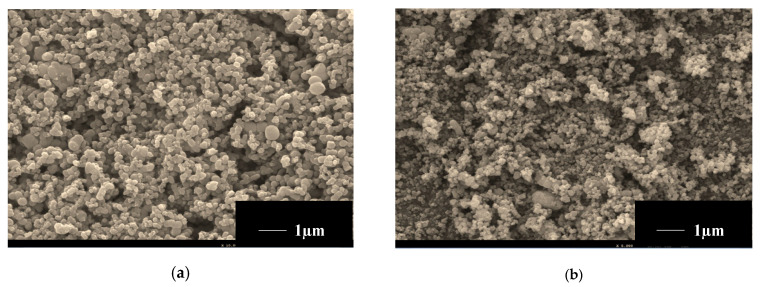
SEM images of: (**a**) Fe_3_O_4_/TiO_2_; (**b**) Fe_3_O_4_/TiO_2_/[FeCl_2_{κ^3^-HC(pz)_3_}].

The non-spherical morphology for the Fe_3_O_4_/TiO_2_ composite (Figure 5a) is probably a result from the high reactivity (very fast hydrolysis) of titanium alkoxide while coating magnetite particles. For Fe_3_O_4_/TiO_2_/[FeCl_2_{κ^3^-HC(pz)_3_}] (Figure 5b) the non-spherical morphology is retained. EDX analysis of this material disclosed the high content of Ti in the shell of particles and confirmed the presence of the C-scorpionate iron(II) complex, as already shown by XPS (Section 3.1).

### 3.4. X-ray Diffraction (XRD)

**Figure 6:**
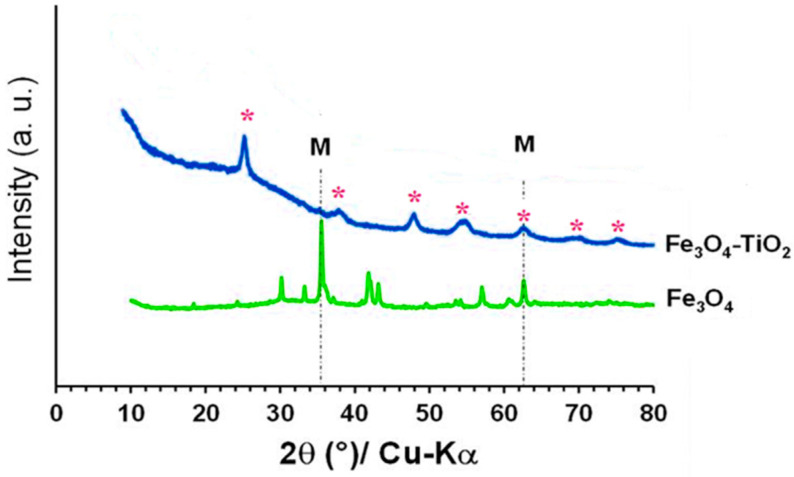
X-ray diffraction profiles of Fe_3_O_4_/TiO_2_ core-shell and its comparison with the industrial magnetite-rich powder; * is anatase (TiO_2_) and M is Fe_3_O_4_.

XRD analysis of the Fe_3_O_4_/TiO_2_ powder indicates the characteristic signals of (TiO_2_) anatase phase (see Figure 6). The peaks at 2-*θ* positions (°) of 25.2, 37.9, 48.0, 54.4, 62.6. 70.0 and 75.5 confirm the formation of a TiO_2_ shell (anatase phase) on the iron oxide. All diffraction peaks were confirmed by literature data [25].

In the XRD profile, very small signals of the highest magnetite peaks (2*θ* = 35.5 and 62.6°) were observed. Similar results were obtained in previous studies for Fe_3_O_4_/TiO_2_ using true pure magnetite sample [26]. XRD signals corresponding to magnetite (core of the systems) are difficult to be detected since a coat of TiO_2_ involving magnetite was formed. It is well known that the shielding or absorption effects by coating structures lead to the weakening of the XRD intensities from the reflections of the core [26]. Core-shell formation is confirmed as these particulate systems have superparamagnetic behavior, and therefore a magnet equally attracts all the particles. These titania-rich particles are attracted under a magnetic field due to their magnetite core.

### 3.5. Fourier Transform Infrared Spectroscopy (FTIR)

FTIR characterization of Fe_3_O_4_/TiO_2_/[FeCl_2_{κ^3^-HC(pz)_3_}] and Fe_3_O_4_/TiO_2_ composites as well as of complex [FeCl_2_{κ^3^-HC(pz)_3_}] was conducted to detect their functional groups. The atom Fe-O vibration was observed at *ν* 422 cm^−1^. Interestingly, in the *ν* 500–700 cm^−1^ range two bond vibrations, Fe-O and Ti-O, of titanium dioxide/magnetite nanocomposite [7,27] were overlapped leading to a broad signal. Bands at *ν* 3386 and 1630 cm^−1^ were assigned to H-O-H vibrations [27] whereas the ones found at *ν* 2876, and 1419 cm^−1^ corresponded to the C-H atom strain vibration. Thus, these results strengthen the above XRD analysis (see Section 3.4) indicating that in the TiO_2_ phase there was an amorphous phase undetectable from the X-ray diffraction peaks.

### 3.6. Catalytic Oxidation of 1-Phenylethanol

**Scheme 1:**
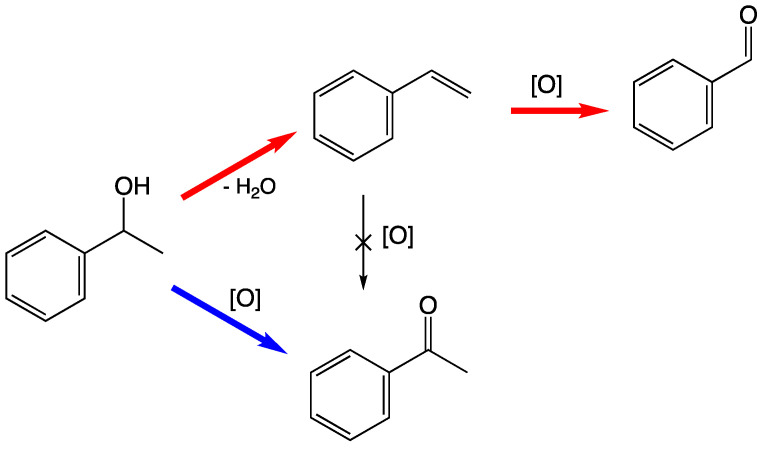
Products from 1-phenylethanol reaction with *tert*-butyl hydroperoxide (TBHP) in the presence of magnetic core-shell Fe_3_O_4_/TiO_2_/[FeCl_2_{κ^3^-HC(pz)_3_}] under different energy inputs (red: conventional thermal heating; blue: all tested inputs).

The solvent-free oxidation of 1-phenylethanol using magnetic Fe_3_O_4_/TiO_2_/[FeCl_2_{κ^3^-HC(pz)_3_}] as heterogeneous catalyst was tested under different energy inputs, i.e., conventional thermal heating, microwave irradiation, mechanical and sonication (see Scheme 1), allowing, for the first time, evaluate the catalytic performance of this new composite in terms of alcohol conversion, product(s) yield, turnover number (TON, number of moles of product per number of moles of catalyst) or turnover frequency (TOF, h^−1^, TON/reaction time).

The catalytic experiments were initiated by choosing, as oxidizing agent, an aqueous solution of *tert*-butyl hydroperoxide (THBP, 70% aqueous solution) and using a very low catalyst (ratio of 1-phenylethanol/Fe: 3200) in solvent-free conditions and using the conventional thermal heating. THBP was preferred to, e.g.*,* hydrogen peroxide in view of its lower handling risk. The main results are presented in Table 1.

The C-scorpionate iron(II) complex supported onto the magnetic core-shell, Fe_3_O_4_/TiO_2_/[FeCl_2_{κ^3^-HC(pz)_3_}], showed high activity as catalyst for the oxidation of 1-phenylethanol under mild conditions. In fact, after only 3 h at 80 °C, 97% conversion of 1-phenylethanol was achieved (entry 3, Table 1), whereas the core-shell Fe_3_O_4_/TiO_2_ under the same conditions led to only 4% of the alcohol conversion (entry 4, Table 1) and control experiments in the absence of any composite led to up to 0.5% conversion. Unexpectedly, besides the usual sole product (acetophenone) formed by 1-phenylethanol oxidation [28,29,30,31], the reaction mixture contained (in lower amount) a product of dehydration of the alcohol–styrene. In addition, benzaldehyde, which is known [32,33,34,35] to be obtained by oxidation of styrene was also found (Scheme 1 and Table 1). To confirm this hypothesis, styrene was used as substrate under the same reaction conditions (entries 11 and 12, Table 1): only benzaldehyde (no acetophenone) was obtained. Thus, the known [18] selectivity (100% for acetophenone) of the C-scorpionate complex was greatly affected by its magnetic core-shell support under the used thermal conditions. A competitive side-reaction is allowed to occur (Scheme 1), lowering the obtained acetophenone yield up to 67% (entry 3, Table 1). This effect was not detected when [FeCl_2_{κ^3^-HC(pz)_3_}] was immobilized at multiwalled carbon nanotubes (MWCNT). The authors reported that 22% of acetophenone (as unique product) was produced after 24 h of oxidation of 1-phenylethanol with TBHP, at 80 °C, in the presence of [FeCl_2_{κ^3^-HC(pz)_3_}]@MWCNT (0.1 mol% vs. alcohol) using an oil-bath heating. They also claim that the low yield of ketone obtained was due to a low substrate consumption rate, while the selectivity was maintained [18]. Moreover, experiments using hydrogen peroxide (30% aqueous solution) as oxidant instead of *tert*-butyl hydroperoxide (70% aqueous solution) resulted in a diminished extent of the competitive dehydration reaction (lower production of secondary products styrene and benzaldehyde; entry 7, Table 1), while led to the same amount of acetophenone (entry 7, Table 1).

**Figure 7:**
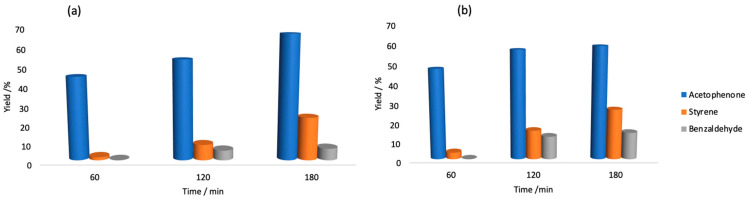
Reaction of 1-phenylethanol to different products (acetophenone, styrene and benzaldehyde) catalyzed by Fe_3_O_4_/TiO_2/_[FeCl_2_{κ^3^-HC(pz)_3_}] under conventional thermal heating, at different reaction times and temperatures: (**a**) 80 °C; (**b**) 120 °C.

It should also be noted that the activity of the studied catalyst increased with reaction time (see entries 1–3 or 8–10, Table 1, Figure 7). Moreover, higher temperatures appear to favor the competitive dehydration reaction, leading to higher contents of the secondary products styrene and benzaldehyde (see entries 1–3 or 8–10, Table 1, Figure 7). Such effect is in agreement with the known [36] harsh conditions usually required for the alcohol to alkene dehydration. On the other hand, performing the reaction at 120 °C also allows to attain faster a higher acetophenone yield, as can be observed in Figure 7. In fact, after the 120 min reaction time, at 120 °C only a residual increase of 3% in the yield of acetophenone is achieved, whereas at 80 °C that increase is of 25% (see entries 2 and 3 or 9 and 10, Table 1).

Importantly, the selectivity to acetophenone, appears also to depend on the Fe_3_O_4_/TiO_2_/[FeCl_2_{κ^3^-HC(pz)_3_}] loading. In fact, an increase of 6 times on the composite amount (from 5.20 × 10^−7^ mol to 3.12 × 10^-6^ mol relative to the C-scorpionate iron(II) complex) results in an acetophenone 99.5% yield increment (from 41.7 to 83.2%), concomitant with a significant reduction on the side-products formation (see entries 3, 5 and 6, Table 1).

**Figure 8:**
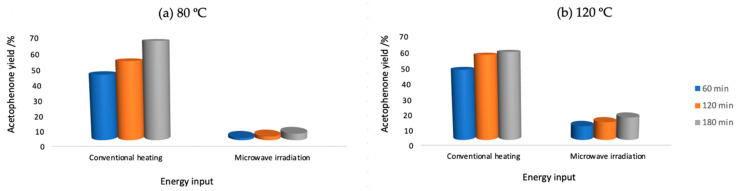
Acetophenone yields obtained from the oxidation of 1-phenylethanol by TBHP, catalyzed by Fe_3_O_4_/TiO_2_/[FeCl_2_{κ^3^-HC(pz)_3_}] under different (conventional thermal heating and microwave irradiation) energy inputs, reaction times (1–3 h) and temperatures: (**a**) 80; (**b**) 120 °C.

The stability of composite Fe_3_O_4_/TiO_2_/[FeCl_2_{κ^3^-HC(pz)_3_}] in the oxidative reaction conditions was evaluated through its potential recyclability up to three consecutive catalytic cycles, as described in Section 2.5, using the conditions of entry 10 of Table 1, i.e., the most harsh conditions used in this study (3 h at 120 °C). As presented in Table 2, under the above-mentioned reaction conditions, there was a drastic catalyst deactivation from the 1st to the 2nd cycle, where a loss of 76% of its initial activity was observed. A consecutive decreasing activity was detected in the 3rd cycle (see Table 2) which prevent further catalytic cycles to be run. Nevertheless, the selectivity to acetophenone increased from the 1st to the 3rd cycle. SEM/EDX analysis of the composite after the 1st cycle (see below, Figure 8a) revealed the occurrence of leaching of the C-scorpionate iron(II) complex from the core-shell surface, that may be responsible for the diminishing of the composite catalytic activity.

The solvent-free microwave-assisted oxidation of 1-phenylethanol by TBHP, catalyzed by Fe_3_O_4_/TiO_2_/[FeCl_2_{κ^3^-HC(pz)_3_}] using the same very low catalyst loading (ratio of 1-phenylethanol/Fe of 3200) was attempted. The main results are presented in Table 3. Under microwave irradiation the catalyst appears to be more selective towards the formation of acetophenone since no by-products were detected by GC-MS. However, significantly better yields of acetophenone were achieved by conventional heating (up to 67% after 3 h at 80 °C, entry 3, Table 1) when compared with microwave irradiation (see Table 3 and Figure 8) which yielded up to 16% of acetophenone after 3 h at 120 °C.

**Figure 9:**
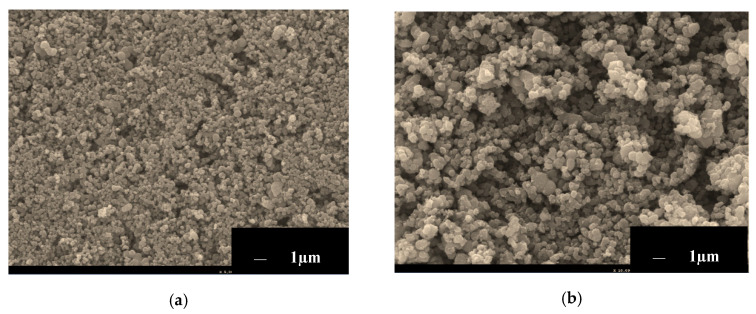
SEM images of magnetic core-shell Fe_3_O_4_/TiO_2_/[FeCl_2_{κ^3^-HC(pz)_3_}]: (**a**) after reaction under conventional thermal heating; (**b**) after reaction under microwave irradiation.

The used (i.e., after the reaction tests) catalysts were characterized by SEM (Figure 9). After the reaction under conventional thermal heating mode (a) the magnetic composite shows a more uniform morphology, while the material that was subject to microwave irradiation (b) clearly shows surface modifications. This change in the morphology during the microwave-assisted reaction could lead to the observed decrease in the catalytic activity. Moreover, EDX analysis showed that the amount of iron at the surface slightly decrease after the reaction, indicating some leaching of the C-scorpionate iron(II) complex from the core-shell surface that may also contribute to the observed diminishing of the composite catalytic activity.

The solvent-free mechanical- or ultrasounds-assisted oxidation of 1-phenylethanol by TBHP, catalyzed by Fe_3_O_4_/TiO_2_/[FeCl_2_{κ^3^-HC(pz)_3_}] using the same very low catalyst loading (ratio of 1-phenylethanol/Fe of 3200) was also performed for comparative purposes. The main results are presented in Table 4. As observed under microwave irradiation, the catalyst in mechano- or sonochemical conditions is selective towards the formation of acetophenone (no by-products were detected by GC-MS). Again, like the microwave-assisted oxidation (Table 3), the mechano- and sonochemical oxidations yielded a significantly lower amount of oxidation product (Table 4) than the formed using thermal heating (Table 1). Smaller reactional times were used in sonication experiments to avoid the occurrence of reactional media (alcohol) evaporation observed for reaction times of 60 or 180 min leading to invalid results. In fact, when the energy from the incident acoustic field is absorbed, is subsequently manifested as a temperature rise whose magnitude is a function of the physical properties of the medium, (e.g., acoustic absorption, density, specific heat), properties of the focused ultrasound device (e.g., beam geometry), and the frequency and time-averaged acoustic intensity of the acoustic field [37].

## 4. Conclusions

To our knowledge, this is the first time that a C-scorpionate complex is immobilized onto a magnetic core-shell support and used as a catalyst for alcohol oxidation under different energy inputs and constitutes an extension of the catalytic application of biologically inspired metal complexes aiming to reach natures’ sustainability. In view of the promising outcomes obtained for the activity of our magnetic core-shell iron(II) C-scorpionate catalyst, further oxidation reactions as well as substrates are planned to be tested.

## Figures and Tables

**Table 1:** Selected catalytic results for the reaction of 1-phenylethanol catalyzed by Fe_3_O_4_/TiO_2_/[FeCl_2_{κ^3^-HC(pz)_3_}] under conventional thermal heating. *^a^*

Entry	t/min	T/°C	Conversion/%	Yield/% *^b^*			Total TON *^c^*	Total TOF/h^−1^ *^d^*
				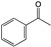	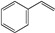	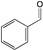		
1	60	80	48.3	44.8	1.6	-	744	744
2	120		69.1	53.8	8.4	5.3	1082	541
3	180		97.1	67.2	23.1	6.4	1548	516
4 *^e^*	180		4.0	2.5	-	-	40	13
5 *^f^*	180		46.9	41.7	5.2	-	2255	752
6 *^g^*	180		96.4	83.2	10.3	2.9	772	257
7 *^h^*	180		83.9	67.3	9.4	5.3	657	219
8	60	120	53.1	47.6	3.5	-	820	820
9	120		95.3	57.4	15.3	12.1	1359	680
10	180		100.0	59.3	26.4	14.2	1601	534
11 *^i^*	120		100.0	-	-	3	37	12
12 *^i^*	180		9.9	-	-	7	86	29

*^a^* General reaction conditions: TBHP (10 mmol), 1-phenylethanol (5 mmol)) and Fe_3_O_4_/TiO_2_/[FeCl_2_{κ^3^-HC(pz)_3_}] (1.56 μmol). *^b^* Yield (%) = moles of product(s) per 100 moles of substrate. *^c^* Turnover number (TON) = moles of product(s) per moles of catalyst. *^d^* Turnover frequency (TOF) (h^−1^) = TON/reaction time. *^e^* Fe_3_O_4_/TiO_2_ (instead of Fe_3_O_4_/TiO_2_/[FeCl_2_{κ^3^-HC(pz)_3_}] as catalyst. *^f^*Fe_3_O_4_/TiO_2_/[FeCl_2_{κ^3^-HC(pz)_3_}] (0.52 μmol). *^g^*Fe_3_O_4_/TiO_2_/[FeCl_2_{κ^3^-HC(pz)_3_}] (3.12 μmol). *^h^*H_2_O_2_ (instead of TBHP) as oxidant. *^i^*Styrene as substrate.

**Table 2:** Catalytic results for the recycling tests of Fe_3_O_4_/TiO_2_/[FeCl_2_{κ^3^-HC(pz)_3_}] under conventional thermal heating. *^a^*

Cycle	Yield/% *^b^*			Total TON *^c^*	Total TOF / h^−1^ *^d^*	Conversion/%
	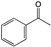	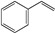	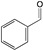			
1st	59.3	26.4	14.2	3202	1067	100.0
2nd	22.4	1.6	-	769	256	25.9
3rd	8.1	-	-	260	87	8.3

*^a^* Reaction conditions: TBHP (10 mmol), 1-phenylethanol (5 mmol) and Fe_3_O_4_/TiO_2_/[FeCl_2_{κ^3^-HC(pz)_3_}] (1.56 μmol), temperature = 80 °C and reaction time = 120 min. *^b^* Yield (%) = moles of product per 100 moles of substrate. *^c^* TON = moles of product per moles of catalyst. *^d^* TOF (h^−1^) = TON/reaction time.

**Table 3:** Selected catalytic results for the oxidation of 1-phenylethanol into acetophenone using, as catalyst, Fe_3_O_4_/TiO_2_/[FeCl_2_{κ^3^-HC(pz)_3_}] under microwave irradiation. *^a^*

Entry	Time/min	Temperature/°C	Yield/% *^b^*	TON *^c^*	TOF/h^−1^ *^d^*	Conversion/%
1	60	80	1.6	51	51	1.8
2	120		2.6	83	42	2.7
3	180		4.6	147	49	4.8
4	60	120	9.5	305	305	10.0
5	120		12.2	391	196	12.2
6	180		15.5	497	166	15.6

*^a^* Reaction conditions: TBHP (10 mmol), 1-phenylethanol (5 mmol) and Fe_3_O_4_/TiO_2_/[FeCl_2_{κ^3^-HC(pz)_3_}] (1.56 μmol). *^b^* Yield (%) = moles of acetophenone per 100 moles of 1-phenylethanol. *^c^* TON = moles of product per moles of catalyst. *^d^* TOF (h^−1^) = TON/reaction time.

**Table 4:** Selected catalytic results for the oxidation of 1-phenylethanol into acetophenone using, as catalyst, Fe_3_O_4_/TiO_2_/[FeCl_2_{κ^3^-HC(pz)_3_}] under two different energy inputs. *^a^*

Entry	Energy Input	Time/min	Yield/% *^b^*	TON *^c^*	TOF/h^−1^ *^d^*	Selectivity/%
1	Mechanical	60	2.5	80	80	96
2		180	6.0	192	64	94
3	Ultrasounds	10	1.7	54	327	98
4		15	3.9	125	500	99

*^a^* Reaction conditions: TBHP (10 mmol), 1-phenylethanol (5 mmol) and Fe_3_O_4_/TiO_2_/[FeCl_2_{κ^3^-HC(pz)_3_}] (1.56 μmol), room temperature; mechanochemistry: 3 spheres, 35 Hz; sonication: 20%. *^b^* Yield (%) = moles of acetophenone per 100 moles of 1-phenylethanol. *^c^* TON = moles of product per moles of catalyst. *^d^* TOF (h^−^^1^) = TON/reaction time.
